# Prognostic implications of valvular heart disease in patients with non-valvular atrial fibrillation

**DOI:** 10.1186/s12872-021-02264-3

**Published:** 2021-09-18

**Authors:** Athanasios Samaras, Eleni Vrana, Anastasios Kartas, Dimitrios V. Moysidis, Andreas S. Papazoglou, Ioannis Doundoulakis, George Fotos, Georgios Rampidis, Dimitrios G. Tsalikakis, Georgios Efthimiadis, Haralambos Karvounis, Apostolos Tzikas, George Giannakoulas

**Affiliations:** 1 1st Department of Cardiology, AHEPA University Hospital, Aristotle University of Thessaloniki, St. Kiriakidi 1, 54636 Thessaloniki, Greece; 2 Department of Informatics and Telecommunication Engineering, University of Western Macedonia, Kozani, Greece; 3 Interbalkan European Medical Center, Thessaloniki, Greece

**Keywords:** Valvular heart disease, Stenosis, Regurgitation, Atrial fibrillation, Prognosis, Outcomes

## Abstract

**Background:**

Valvular heart disease (VHD) in non-valvular atrial fibrillation (AF) is a puzzling clinical entity. The aim of this study was to evaluate the prognostic effect of significant VHD (sVHD) among patients with non-valvular AF.

**Methods:**

This is a post-hoc analysis of the MISOAC-AF trial (NCT02941978). Consecutive inpatients with non-valvular AF who underwent echocardiography were included. sVHD was defined as the presence of at least moderate aortic stenosis (AS) or aortic/mitral/tricuspid regurgitation (AR/MR/TR). Cox regression analyses with covariate adjustments were used for outcome prediction.

**Results:**

In total, 983 patients with non-valvular AF (median age 76 [14] years) were analyzed over a median follow-up period of 32 [20] months. sVHD was diagnosed in 575 (58.5%) AF patients. sVHD was associated with all-cause mortality (21.6%/yr vs. 6.5%/yr; adjusted HR [aHR] 1.55, 95% confidence interval [CI] 1.17–2.06; *p* = 0.02), cardiovascular mortality (16%/yr vs. 4%/yr; aHR 1.70, 95% CI 1.09–2.66; *p* = 0.02) and heart failure-hospitalization (5.8%/yr vs. 1.8%/yr; aHR 2.53, 95% CI 1.35–4.63; *p* = 0.02). The prognostic effect of sVHD was particularly evident in patients aged < 80 years and in those without history of heart failure (*p* for interaction < 0.05, in both subgroups). After multivariable adjustment, moderate/severe AS and TR were associated with mortality, while AS and MR with heart failure-hospitalization.

**Conclusion:**

Among patients with non-valvular AF, sVHD was highly prevalent and beared high prognostic value across a wide spectrum of clinical outcomes, especially in patients aged < 80 years or in the absence of heart failure. Predominantly AS, as well as MR and TR, were associated with worse prognosis.

**Supplementary Information:**

The online version contains supplementary material available at 10.1186/s12872-021-02264-3.

## Highlights

- The puzzling entity of valvular heart disease in non-valvular atrial fibrillation is being reevaluated in recent guidelines
- Moderate/severe valvular heart disease was associated with all-cause death, cardiovascular death and heart failure-hospitalization, and its prognostic effect was particularly evident in patients aged < 80 years old and in those without history of heart failure
- Individual moderate/severe valve lesions (predominantly aortic stenosis) were predictive of death and heart failure-hospitalization but not stroke/SEE or major bleeding.
- The generally accepted term “non-valvular AF” is
  i. A misnomer, since almost 60% of patients with non-valvular atrial fibrillation appeared to have moderate/severe valvular heart disease
  ii. Misleading and confusing in daily clinical practice, given the increased use of VKAs, compared with NOACs, in patients with moderate/severe valvular heart disease

## Introduction

Atrial fibrillation (AF) and valvular heart disease (VHD) are frequently encountered in clinical practice, and often coexist, especially in the elderly population [1–3]. Both conditions are associated with increased mortality and morbidity [4–6]. Recent guidelines suggest careful evaluation of patients with AF and VHD due to the puzzling nature of their coexistence [7–9].

Post-hoc sub-analyses of the existing randomized controlled trials on oral anticoagulation have demonstrated the prognostic significance of VHD across a plethora of outcomes among patients with AF [10–15]. Valve lesions have been compared on the basis of their association with specific outcomes, revealing contradictory results between studies [11, 13, 16]. However, the predictive performance of VHD in AF outside highly-selected trial cohorts remains debatable, since outcomes have been mainly analyzed under the scope of comparisons between type and dosage of oral anticoagulants [19,20,21].

The main objectives of this study were to evaluate the association of sVHD and individual valve lesions with clinical outcomes, and to detect specific patient subgroups where the prognostic value of sVHD is particularly evident.

## Methods

### Study population

This is a post-hoc analysis of the MISOAC-AF trial [22, 23]. This study population comprised consecutive adult patients who were hospitalized in the Cardiology ward from December 2015 to June 2018 with any diagnosis and comorbid AF. Atrial fibrillation was defined as previously documented in the medical record or new-onset AF during hospitalization detected by a 12-lead electrocardiogram/24-h Holter monitoring [9]. Patients with moderate/severe mitral stenosis and those with mechanical prosthetic heart valve were considered to have “valvular AF” [9]. Patients with valvular AF, unavailable echocardiographic data or life expectancy < 6 months were excluded from the present study.

### Echocardiographic assessment

All patients included in this study had a transthoracic echocardiography available for analysis during their hospitalization. Echocardiographic studies were performed by trained experienced cardiologists. Parasternal, apical, and subcostal views were used to acquire M-mode and 2D-dimensional, color, pulsed and continuous wave Doppler data. Left ventricular ejection fraction (LVEF) was evaluated by the difference between LV end-diastolic and end-systolic volumes relative to the LV end-diastolic volume [24]. Left atrial volume was measured in the apical 4- and 2-chamber views using Simpson’s biplane method of discs and was indexed (LAVi) to the body surface area of each patient [24]. The presence and severity of valve lesions was based on recent guidelines [7, 8].

Patients were considered to have sVHD if they had echocardiographic evidence of at least moderate AS, AR, MR, or TR. Minimal paravalvular leaks and loose narrowings were not considered as significant valve regurgitation and stenosis, respectively, and were therefore both categorized as no/mild VHD [25]. Individuals with a history of bioprosthetic valve placement were not considered to have sVHD, unless the echocardiography during hospitalization revealed moderate/severe valve lesions.

### Follow-up and outcomes

Outcome data were obtained until July 2020. All patients were followed up for the clinical outcomes of all-cause or cardiovascular mortality, stroke or systemic embolic event (SEE), major bleeding and re-hospitalization. Further analyses was done based on net clinical outcomes of (1) cardiovascular death or stroke/SEE or major bleeding, (2) cardiovascular death or HF hospitalization, (3) cardiovascular death or any hospitalization, and (4) all-cause death or HF hospitalization. Updated information of vital status of all patients was obtained by the Greek Civil Registration System, and was additionally verified and classified by registration data, inpatient hospital records, death certificates and telephone contact with retirement homes or families. Telephone and in-person contacts at 6-month intervals after the initial hospital discharge were performed for evaluation of clinical outcomes. Blinded physicians reviewed and adjudicated the outcome events, through a thorough examination of all available follow- up sources. Cardiovascular mortality was defined as death where cardiovascular disease was reported as the underlying cause of death, including sudden cardiac death, or death due to heart failure, acute coronary syndrome, pulmonary embolism, stroke, hemorrhage, or other cardiovascular causes [26]. Major bleeding was defined according to the International Society on Thrombosis and Haemostasis [27].

### Statistical analysis

Continuous variables were tested for normality, and presented as means with standard deviations (SD) or medians with interquartile range (IQR), with comparisons made using the Student's t-test or the Mann–Whitney U. Categorical variables are expressed as frequencies (%), with comparisons made using the Pearson’s χ^2^ test.

Time-to-event was estimated using the Kaplan–Meier method and the time-to-event rates were compared across groups with log-rank tests. Cox proportional hazards models were used to test the association between sVHD or moderate/severe valve lesions with clinical outcomes. Multivariable models were adjusted for variables, on the basis of their prognostic significance when tested univariately and their clinical relevance to the study outcomes. Specifically, adjustments were performed for the following variables: age, gender, body mass index, AF pattern, duration of AF, heart failure, chronic obstructive pulmonary disease, coronary artery disease, prior myocardial infarction, prior stroke, prior major bleeding, estimated glomerular filtration rate, LVEF, LAVi, N-terminal pro B-type natriuretic peptide, high-sensitive cardiac troponin T, type of oral anticoagulation and rate or rhythm control strategy. Proportional hazards assumptions were assessed by plotting the log–log Kaplan–Meier curves; no violations were observed. The results were expressed as hazard ratios (HR) and 95% confidence intervals (CI).

Subgroup analyses were performed to evaluate potential discrepancies in the association of sVHD with clinical outcomes across patient subsets of interest, including age, gender, AF pattern, heart failure, LVEF, coronary artery disease, estimated glomerular filtration rate, oral anticoagulation and pulmonary regurgitation. *p* values for the interaction are therefore provided. A two-sided *p* value of less than 0.05 was accepted as statistically significant. Statistical analysis was performed using SPSS v24 (SPSS Inc., Chicago, Illinois) and Stata v15.1 (StataCorp, College station, Texas, United States) packages.

This study was approved by the institutional research ethics committee (Aristotle University of Thessaloniki Research Ethics Committee).

## Results

### Patients

**Fig. 1:**
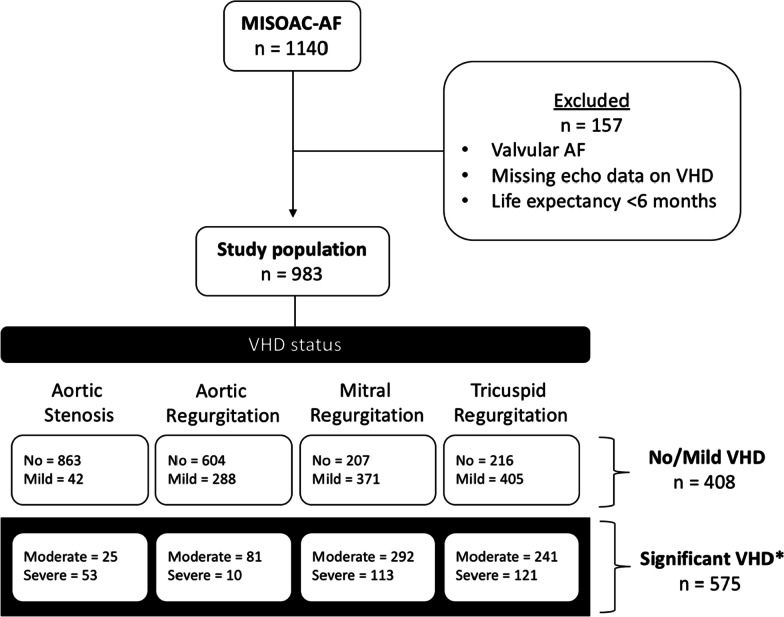
Flowchart of patient population**.** VHD, valvular heart disease. *Significant VHD represents moderate/severe valve lesions. The categories of VHD are not mutually exclusive. Patients with significant VHD may have multiple moderate/severe valve lesions, while patients with no/mild VHD may have multiple mild valve lesions or no VHD

In the MISOAC-AF trial, 1140 consecutive patients with AF were initially screened. Excluding those with valvular AF, unavailable echocardiographic data or life expectancy < 6 months, a total of 983 patients were included in the present study. The flowchart of the study population is presented in Fig. 1. Among the 983 patients, moderate/severe valve lesions were identified as follows: AS is 78 patients (7.9%), AR in 91 (9.3%), MR in 405 (41.2%) and TR in 362 (36.8%). sVHD was diagnosed in 575 (58.5%) of the patients, while the rest had mild/no VHD. The categories of VHD are not mutually exclusive.

### Baseline characteristics

Demographics, baseline characteristics, medical history and discharge medication of the whole population, as well as of patients with or without sVHD are described in Table 1. Compared with patients with no/mild VHD, patients with sVHD were older, more often women, had higher thromboembolic and bleeding risks, and greater prevalence of comorbidities. Moreover, patients with sVHD were treated more often with VKAs (compared with NOACs), beta-blockers, diuretics and aldosterone receptor antagonists, and less often with antiarrhythmic agents and ACE inhibitors/ARBs.

**Table 1:** Characteristics of patients according to VHD status

Variables	All^1^ (n = 983)	No/mild VHD^a^ (n = 408)	Significant VHD^a^ (n = 575)		*p* value^b^
Age (years)	76 (14)	72 (16)	78 (11)		** < 0.001**
Gender (men)	553 (54.2)	243 (59.6)	290 (50.4)		**0.005**
BMI (kg/m^2^)	28 (6)	29 (6)	25 (3)		**0.003**
*CHA*_*2*_*DS*_*2*_*-VASc score*					
Mean (SD)	4.3 ± 2.0	3.8 ± 2.0	4.7 ± 1.9		** < 0.001**
Median (IQR)	4 (3)	4 (2)	5 (2)		** < 0.001**
*HASBLED score*					
Mean (SD)	1.9 ± 1.1	1.7 ± 1.1	2.0 ± 1.1		** < 0.001**
Median (IQR)	2 (2)	2 (1)	2 (2)		** < 0.001**
*AF pattern*					
First-diagnosed	138 (14)	71 (17.4)	67 (11.7)		** < 0.001**
Paroxysmal	364 (37)	198 (48.5)	166 (28.9)		** < 0.001**
Persistent or permanent	481 (48.9)	139 (34.1)	342 (59.5)		** < 0.001**
Duration of AF (years)	4.0 (9.9)	3.0 (6.98)	4.2 (9.8)		** < 0.001**
*Clinical history*					
Hypertension	788 (80.2)	325 (79.7)		463 (80.5)	0.738
Diabetes mellitus	339 (34.5)	128 (31.4)		211 (36.7)	0.084
Hyperlipidemia	485 (49.3)	198 (48.5)		287 (49.9)	0.669
Heart failure	483 (49.1)	128 (31.4)		355 (61.7)	** < 0.001**
Endocrinal disease	221 (22.5)	94 (23)		127 (22.1)	0.725
COPD	132 (13.4)	41 (10)		91 (15.8)	**0.009**
Coronary artery disease	429 (43.6)	161 (39.5)		268 (46.6)	**0.026**
Prior myocardial infarction	239 (24.3)	80 (19.6)		159 (27.7)	**0.004**
Prior PCI or CABG	278 (32.3)	126 (30.9)		192 (33.4)	0.407
Prior cardiac arrest	26 (2.6)	9 (2.2)		17 (3)	0.470
Non-fatal stroke or SEE	147 (15)	59 (14.5)		88 (15.3)	0.715
Non-fatal major hemorrhage	154 (15.7)	46 (11.3)		108 (18.8)	**0.001**
Bioprosthetic valve	16 (1.6)	4 (1.0)		12 (2.1)	0.206
eGFR at discharge (ml/min/1.73m^2^)	60 (40)	71 (49)		55 (34)	** < 0.001**
LVEF (%)	52 (14)	55 (10)		50 (15)	** < 0.001**
LAVi (mL/m^2^)	41 (11)	37 (13)		44 (10)	** < 0.001**
NT-proBNP (pg/ml)	2047 (4497)	1422 (2930)		2771 (5746)	** < 0.001**
hs-TnT (pg/ml)	27 (43)	20 (32)		34 (51)	** < 0.001**
*Medication at discharge*					
Oral anticoagulants	781 (79.4)	312 (76.5)	469 (81.6)		0.242
Vitamin K antagonist	257 (26.1)	81 (19.9)	176 (30.6)		** < 0.001**
Non-vitamin K antagonist	524 (53.3)	231 (56.6)	293 (51)		**0.02**
Antiplatelet agent	265 (27)	109 (27)	156 (27.1)		0.885
Aspirin	102 (10.4)	37 (9.1)	65 (11.3)		0.257
Clopidogrel	60 (6.1)	26 (6.4)	34 (5.9)		0.767
Dual antiplatelet	103 (10.5)	46 (11.3)	57 (9.9)		0.492
B-blocker	739 (75.2)	289 (70.8)	450 (77.3)		**0.005**
Digoxin	60 (6.1)	17 (4.1)	48 (8.3)		**0.009**
Calcium channel blocker	204 (20.8)	95 (23.3)	109 (19)		0.137
Antiarrhythmic agent	228 (23.2)	117 (30.1)	105 (18.3)		** < 0.001**
Amiodarone	178 (18.1)	95 (23.3)	83 (14.4)		** < 0.001**
Propafenone	27 (2.7)	18 (4.4)	9 (1.6)		**0.007**
Sotalol	23 (2.3)	10 (2.5)	13 (2.3)		0.846
ACE inhibitors or ARBs	429 (43.6)	195 (47.8)	234 (40.7)		**0.046**
MRAs	257 (26.1)	66 (16.2)	191 (33.2)		** < 0.001**
Statins	387 (39.4)	175 (42.9)	212 (36.9)		0.089
Diuretics	591 (60.1)	181 (44.4)	410 (71.3)		** < 0.001**

^a^Data were reported as absolute numbers (%), means (SD), or medians (IQR)

^b^Values in bold indicate statistical significance (*p* < 0.05)

*VHD* valvular heart disease, *AF* atrial fibrillation, *BMI* body mass index, *eGFR* estimated glomerular filtration rate, *COPD* chronic obstructive pulmonary disease, *PCI* percutaneous coronary intervention, *CABG* coronary artery by-pass, *SEE* systemic embolic event, *LVEF* left ventricular ejection fraction, *LAVi* indexed left atrial volume, *NT-proBNP* N-terminal pro B-type natriuretic peptide, *hs-TnT* cardiac troponin T measured with high-sensitivity assay, *ACE* angiotensin-converting enzyme, *ARB* angiotensin receptor blocker, *MRA* mineralocorticoid receptor antagonists

### Outcomes according to VHD status

**Fig. 2:**
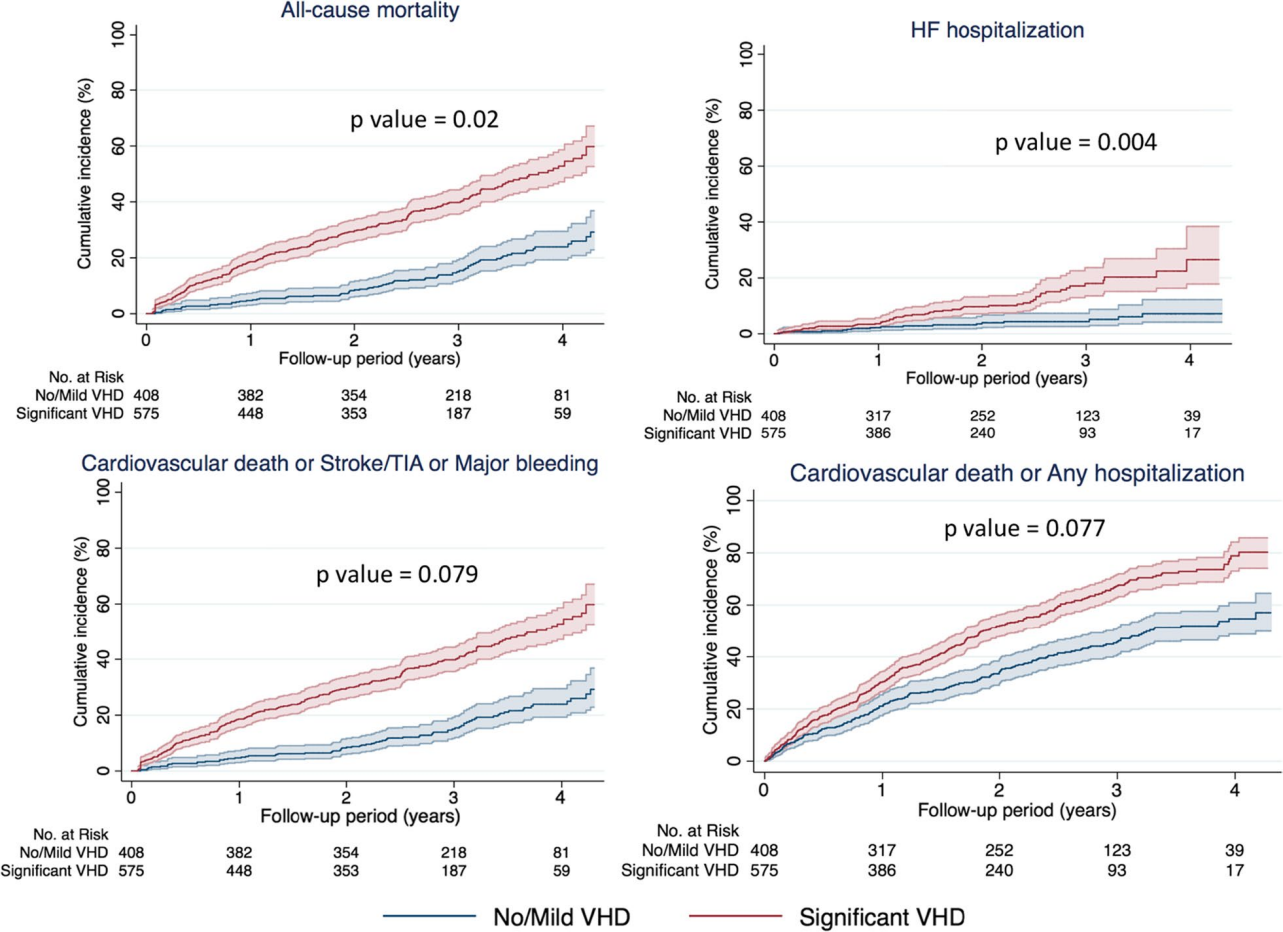
Cumulative incidence of clinical outcomes by VHD status. Each curve is accompanied by lines representing 95% confidence intervals. VHD, valvular heart disease; HF, heart failure; SEE, systemic embolic event

During a median follow-up period of 32 months (IQR 20, max 56), the presence of sVHD was associated with all-cause mortality (21.6%/yr vs. 6.5%/yr; adjusted HR [aHR] 1.55, 95% confidence interval [CI] 1.17–2.06; *p* = 0.02), cardiovascular mortality (16%/yr vs. 4%/yr; aHR 1.70, 95% CI 1.09–2.66; *p* = 0.02) and heart failure-related hospitalization (5.8%/yr vs. 1.8%/yr; aHR 2.53, 95% CI 1.35–4.63; *p* = 0.02) (Table 2). Stroke/SEE and major bleeding did not differ between VHD status. All net clinical outcomes of interest occurred more frequently in sVHD than in no/mild-VHD patients (Table 2). Kaplan–Meier curves for outcomes by VHD status are shown in Fig. 2.

**Table 2:** Clinical outcomes according to VHD status

Study Outcomes	Significant VHD (n = 575)		No/mild VHD (n = 408)		Unadjusted		Adjusted^c^	
	n	Incidence rate^1^ (per 100 pt-yrs)	n	Incidence rate^a^ (per 100 pt-yrs)	HR (95% CI)^b^	p value^d^	HR (95% CI)^b^	*p* value^d^
All-cause death	277	21.6	79	6.5	3.24 (2.52–4.16)	** < 0.001**	1.55 (1.17–2.06)	**0.02**
Cardiovascular death	208	16.0	49	4.0	3.87 (2.83–5.29)	** < 0.001**	1.70 (1.09–2.66)	**0.02**
Stroke/systemic embolic event	25	2.1	14	1.2	1.77 (0.92–3.43)	0.089	1.80 (0.83–3.91)	0.136
Major bleeding	22	1.9	22	2.0	0.88 (0.49–1.59)	0.669	0.60 (0.29–1.24)	0.167
HF hospitalization	58	5.8	17	1.8	4.08 (2.37–7.04)	** < 0.001**	2.53 (1.35–4.73)	**0.004**
Any hospitalization	202	20.3	155	16.9	1.48 (1.20–1.84)	** < 0.001**	1.18 (0.92–1.52)	0.202
*Net clinical outcomes*								
Cardiovascular death or stroke/SEE or major bleeding	239	22.9	81	8.8	2.72 (2.11–3.51)	** < 0.001**	1.30 (0.97–1.74)	0.079
Cardiovascular death or HF hospitalization	249	24.5	62	6.7	3.67 (2.78–4.85)	** < 0.001**	1.74 (1.19–2.55)	**0.005**
Cardiovascular death or Any hospitalization	369	36.6	192	20.9	1.76 (1.48–2.09)	** < 0.001**	1.20 (0.98–1.47)	0.077
All-cause death or HF hospitalization	316	31.3	92	9.9	3.17 (2.51–4.01)	** < 0.001**	1.61 (1.24–2.08)	** < 0.001**

^a^Incidence rates are expressed per 100 patient-years. ^b^Hazard ratios and 95% CI are presented using a Cox proportional hazards regression. ^c^Adjusted hazard ratio indicates adjustment for variables that were individually associated with each outcome, including age, gender, body mass index, AF pattern, duration of AF, heart failure, chronic obstructive pulmonary disease, coronary artery disease, prior myocardial infarction, prior stroke, prior major bleeding, estimated glomerular filtration rate, LVEF, LAVi, N-terminal pro B-type natriuretic peptide, high-sensitive cardiac troponin T, type of oral anticoagulation and rate or rhythm control strategy. ^d^Values in bold indicate statistical significance (*p* < 0.05)

*VHD* valvular heart disease, *SEE* systemic embolic event, *HF* heart failure, *CI* confidence interval

### Outcomes according to specific valve lesions

**Fig. 3:**
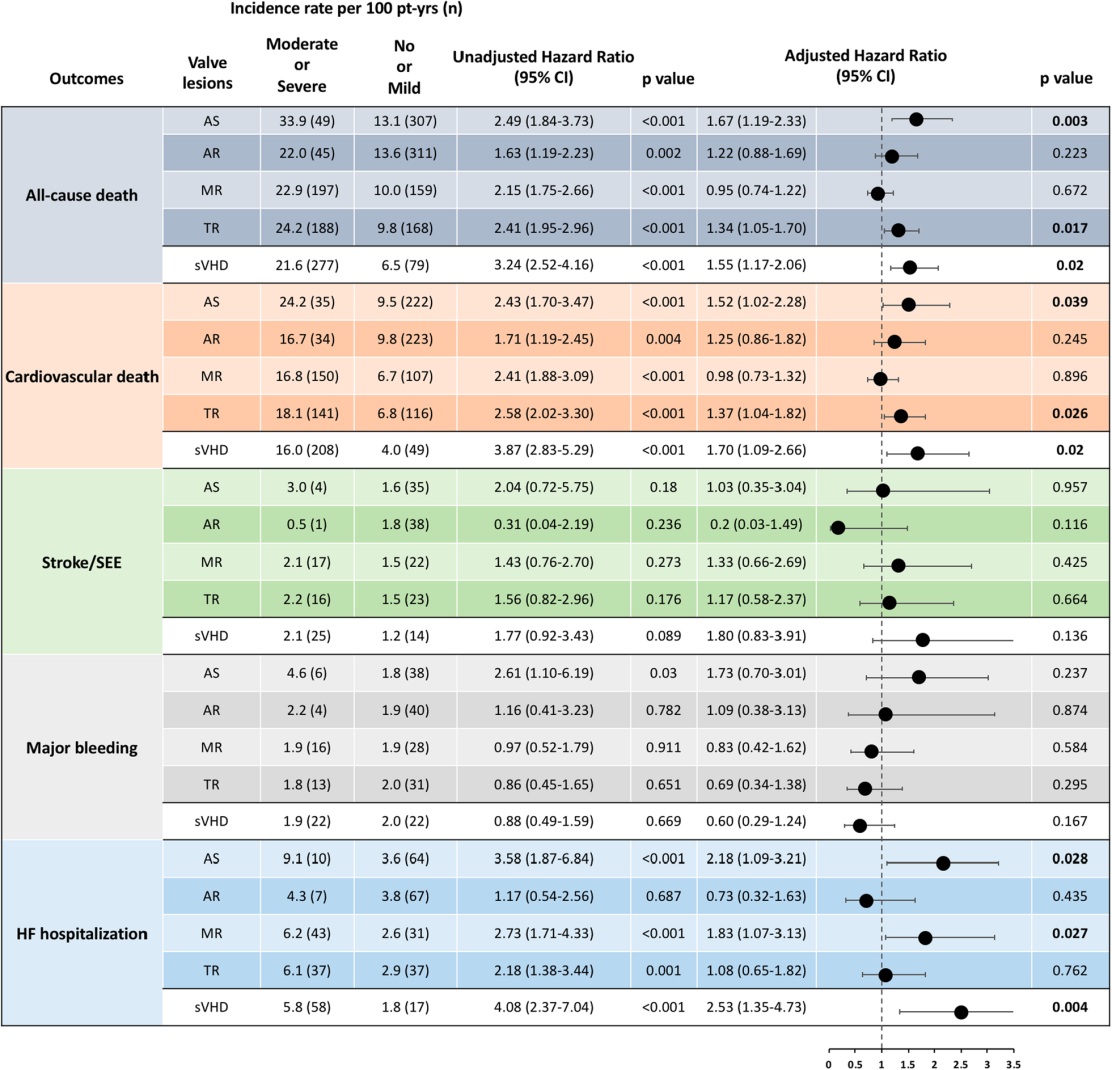
Prognostic association of moderate/severe valve lesions across clinical outcomes. Incidence rates, unadjusted and adjusted hazard ratios are presented. Adjustments were done similarly to Table 2, as well as for other valve lesions with significant association to each outcome. *AS* aortic stenosis, *AR* aortic regurgitation, *MR* mitral regurgitation, *TR* tricuspic regurgitation, *HR* hazard ratio, *CI* confidence interval, *SEE* systemic embolic event, *HF* heart failure, *sVHD* significant valvular heart disease

Moderate/severe AS, AR, MR, TR were all individually associated with all-cause and cardiovascular mortality (Fig. 3). After adjustment for clinical variables that significantly contributed to prediction of each outcome, only AS and TR appeared to maintain their significant association with death. Furthermore, AS and MR appeared to be independent predictors of HF-hospitalization (Fig. 3). Among valve lesions, only AS had an independent and graded (see Additional file 1) association with net clinical outcomes.

### Subgroup analysis

**Fig. 4:**
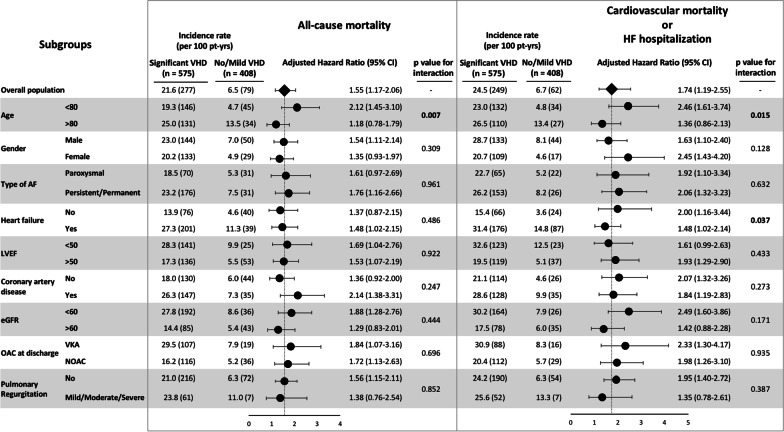
Major subgroup analyses of all-cause mortality and cardiovascular mortality/HF hospitalization by VHD status. *p* values for interaction across subgroups were presented. Hazard ratios and 95% confidence intervals were adjusted for covariates. The prognostic value of VHD was emphasized in patients < 80 years old for both clinical outcomes, and in the absence of heart failure for prediction of cardiovascular mortality/HF hospitalization. Abbreviations as previously reported

**Fig. 5:**
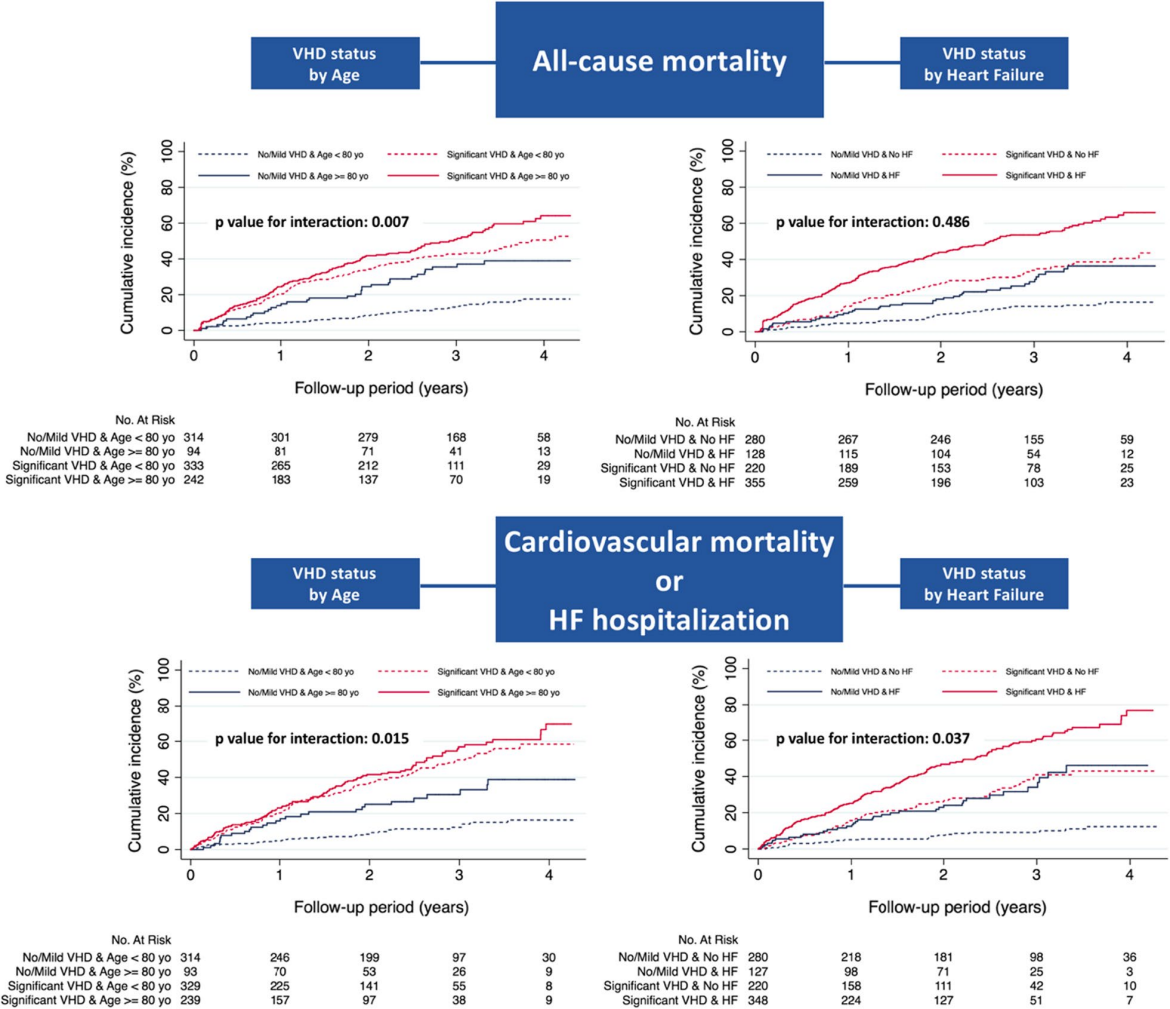
Cumulative incidence of all-cause mortality and cardiovascular mortality/HF hospitalization by VHD status and age or history of heart failure. The prognostic effect of significant VHD is particularly evident in patients < 80 years old and in those without history of heart failure, as indicated by the *p* values for interaction across subgroups. VHD, valvular heart disease; HF, heart failure

Major subgroup analyses were performed according to age, gender, AF pattern, heart failure, LVEF, coronary artery disease, eGFR, type of OAC, and pulmonary regurgitation (Fig. 4). The association of VHD with all-cause mortality and CV mortality/HF hospitalization was consistent across subgroups after multivariate adjustment (aHR > 1). In patients aged < 80 years old the presence of sVHD appeared to have significantly more prognostic value for all-cause mortality (aHR 2.12 vs. 1.18; *p* for interaction = 0.007) and CV mortality/HF hospitalization (aHR 2.46 vs. 1.36; *p* for interaction = 0.015), compared with patients aged > 80 years old. The benefit of sVHD in predicting CV mortality/HF hospitalization was also increased in the absence of heart failure (aHR 2.00 vs. 1.48; *p* for interaction = 0.037). Kaplan–Meier curves for all-cause mortality and CV mortality/HF hospitalization according to VHD and age or history of heart failure are displayed in Fig. 5.

## Discussion

In this post-hoc analysis of the MISOAC-AF clinical trial, comprising a relatively multimorbid patient population with non-valvular AF, several findings were noted: (1) moderate or severe VHD (sVHD) accounted for almost 6 out of 10 patients with non-valvular AF, while patients with sVHD and non-valvular AF received significantly more VKAs compared with NOAC, (2) sVHD was an independent predictor of mortality and HF-related hospitalization; (3) the prognostic value of sVHD was particularly evident in patients aged < 80 years old and in those without history of heart failure; and (4) moderate/severe AS and TR were both associated with all-cause and cardiovascular mortality, while moderate/severe AS and MR were associated with HF-hospitalization.

Five post-hoc analyses of large randomized clinical trials (RCTs) on oral anticoagulation (ENGAGE AF-TIMI [2017], ORBIT-AF [2017], RE-LY [2016], ARISTOTLE [2015], ROCKET-AF [2014]) have analyzed the prevalence and prognostic value of VHD in patients with AF [10–14]. To place these pivotal trials into perspective, the prevalence of sVHD (moderate/severe VHD) was 13% (n = 2824) in ENGAGE AF-TIMI, 27.7% (n = 2705) in ORBIT-AF, 21.8% (n = 3950) in RE-LY, 26.4% (n = 4808) in ARISTOTLE and 14.1% (n = 2003) in ROCKET-AF. In our study, 58.5% (n = 575) had sVHD, which is remarkably higher compared with the aforementioned studies. This discrepancy has three main etiologies; (1) the higher rate of comorbidities of our patient population, (2) the strict eligibility criteria of the RCTs compared with the MISOAC-AF trial, and (3) the varying definitions of sVHD across studies. Indeed, our study included only AF inpatients who were hospitalized for cardiac reasons [28], which is undoubtedly a relatively multimorbid group of patients. This is additionally confirmed by the higher age (median of 76 [14]) and CHA2DS2-VASc score (mean of 4.3) of our patients, compared to other AF registries. Furthermore, the less strict inclusion/exclusion criteria of our study [22], compared with other large trials, extended the range of included patients. Thus, our study could be a better reflection of “real-world” patients with AF, and our results could be indicative of the true prevalence of VHD among patients with non-valvular AF. Lastly, our study also included patients with TR, which was highly prevalent in our population (36.8%). This could partially explain the big difference in sVHD prevalence between our and other studies such as the ROCKET-AF and ENGAGE-AF, which did not include TR in their definition of sVHD.

In the ENGAGE AF-TIMI 48 study, the presence of VHD significantly increased the risk for all-cause (aHR 1.40) and cardiovascular death (aHR 1.47), major bleeding (aHR 1.21), major adverse cardiac events (aHR 1.29), as well as composite endpoints of stroke/SEE or death (aHR 1.30) [10]. However, the outcomes of stroke/SEE were similar in AF patients with and without VHD [10]. In the ORBIT-AF study, VHD was significantly associated with all-cause death (aHR 1.23), while stroke and major bleeding were not related with VHD status after adjustment for covariates. In the RE-LY and ROCKET-AF studies, major bleeding was the only outcome related with VHD status (aHR 1.32 in both studies). Contrarily, in the ARISTOTLE study, patients with VHD had higher rates of stroke/SSE (aHR 1.34) and death (aHR 1.48), but similar rates of major bleeding.

The aforementioned results reveal a lack of homogeneity across studies regarding the prognostic effect of VHD on clinical outcomes. A recent meta-analysis that analyzed patients enrolled in these studies reported that patients with VHD had higher risks of mortality and major bleeding but not stroke or SEE [21]. In our study, sVHD was significantly associated with all-cause and cardiovascular mortality, which is in agreement with results from the ENGAGE AF-TIMI, ORBIT AF and ARISTOTLE studies. No association was noted between VHD status and risk of stroke/SEE in our study, results that were consistent with four out of five studies. However, the analysis of the risk of major bleeding in our study produced conflicting results with those of other studies, since it appeared to be similar between VHD groups, and even numerically reduced in patients with VHD. The reasons for this specific finding are unknown, and not explained by the higher baseline bleeding risk of the VHD subgroup. Interestingly, the risk of HF-hospitalization, which is an outcome that was not examined in previous studies, appeared to be 2.5 times greater in patients with sVHD in our study. This observation has several implications regarding increase in health care costs, and warrants further investigation in future studies.

Of note, the prognostic effect of sVHD was particularly evident in patients < 80 years old and in those without history of heart failure. This could be partially explained by the fact that older patients or patients with HF have competing risks for worse outcomes due to multimorbidity; hence, sVHD is being heavily competed by other clinical factors (frailty, cancer, etc.). As a result, the presence of sVHD has an increased impact on clinical outcomes and becomes more evident/profound in “younger, HF-free” patients. Even though results were adjusted for covariates, it is likely that some residual confounding still exists. To our knowledge, this is the first subgroup analysis in patients with VHD and AF. Thus, careful evaluation of the presence and severity of VHD in these specific subgroups and modifications in the management of these patients is encouraged to ultimately improve prognosis.

The association of individual valve lesions with clinical outcomes has been inadequately investigated in patients with AF. Few studies have reported on the prognostic value of AS [11, 18, 29]. A sub-analysis of the ROCKET-AF trial [29] and a Danish nationwide study [18] compared AS with AR and MR, showing its prognostic superiority for all-cause death, stroke/SEE and major bleeding. In a post-hoc analysis of the ORBIT-AF trial, AS was associated with higher risk of death, but not stroke or major bleeding [11]. Data from our study suggest that AS has a graded and independent association with increased risk of all-cause death, cardiovascular death and several net clinical outcomes. Patients with moderate/severe AS had 1-year mortality risk of 33.9%, which is considerably higher compared with cohorts of patients with [18] or without AF [30]. Stroke/SEE and major bleeding events did not differ based on VHD status in our study. However, the risk of HF-hospitalization was more than two-fold in patients with AS, which has not been reported in previous studies. AR did not have any association with clinical outcomes neither in our study, nor in other registries [11, 18, 29]. MR appeared to be the most prevalent valve lesion across AF studies [21], even though it has not been showed conflicting results when analyzed. Specifically, recent results associate MR with higher risks of all-cause and cardiovascular death [31] but not thromboembolic events [18, 31], which is contradictory to earlier studies that reported the protective effect of MR against stroke [32, 33]. In our registry, MR was indeed the most prevalent valve lesion, while patients with moderate/severe MR were at higher risk of HF-hospitalization but not death, stroke/SEE or major bleeding. The prognostic effect of TR has not been analyzed in patients with AF, since large AF trials did not include TR in their definition of VHD due to its unlikely impact on thromboembolic risk. Results from our study suggest that moderate/severe TR was an independent predictor of all-cause and cardiovascular death, beside AS, indicating the need for careful evaluation of patients with AF and TR.

Two particularly obvious, yet very interesting, observations should be underlined that warrant a careful evaluation of patients with valvular heart disease. Firstly, the results of this study suggest that the generally accepted term “non-valvular AF” is a misnomer, since more than half of our patient population with non-valvular AF had significant VHD, even after exclusion of those with mitral stenosis or mechanical heart valves. Secondly, the so-called “non-valvular AF” could also be largely misleading and confusing in daily clinical practice, as demonstrated by the fact that significantly more patients with sVHD that could benefit from the use of NOACs [9, 34] were treated with VKAs in our cohort, in contrast to patients without sVHD in whom NOACs were preferred. Currently, there is no evidence that the presence of VHD, other than moderate-to-severe MS and mechanical prosthetic heart valves, should modify the choice of oral anticoagulation [35]. However, as indicated in our study the current labeling and description of these agents as being indicated for non-valvular AF may be leading to undertreatment of patients with VHD. These observations have also been stressed out in a paucity of studies [13, 14, 19, 35], which ultimately led to a suggestion for abandonment of the terms “valvular/non-valvular AF” in the recent 2020 AF guidelines [9].

## Strengths and limitations

This study has a number of strengths. It is the first one that examined the risk of HF-hospitalization according to VHD status. The study is also unique because of the subgroup analyses, and more importantly the identification of specific subgroups of clinical interest in which the prognostic effect of VHD becomes more evident. Moreover, the study explored the association of individual valve lesions with several outcomes, which has not been adequately investigated in previous studies. Finally, it could be argued that the patients of this study represented more accurately a “real-world” AF population, since the MISOAC-AF trial avoided the strict eligibility criteria of other large RCTs.

As for the study’s limitations, this post-hoc analysis in patients with and without VHD was not prespecified. The number of cases for some events were too small, which may have weakened the validity of Cox regression analysis. The medium sized sample resulted in smaller subgroups; thus, interpretation should be considered in this context. However, the high event rates and the large proportion of patients with VHD likely provided adequate statistical power to detect heterogeneity across subgroup analyses. There was no information on the cause of valve lesions. Patients had substantial differences in baseline characteristics based on VHD status, and even though multivariable adjustment was performed, some residual confounding likely still exists. Comparisons of risks of events in patients treated with either VKA or NOAC cannot be made since this study was not designed for this.

## Conclusions

In this contemporary registry of patients with non-valvular AF that were hospitalized in the Cardiology ward, almost 60% had moderate/severe VHD. VHD was associated with mortality and HF-hospitalization and its prognostic effect was particularly evident in patients aged < 80 years old and in those without history of heart failure. Moderate/severe AS had the greatest prognostic value among valve lesions. Increased use of VKAs was noted in patients with VHD, which implied that the term “non-valvular AF” might be confusing and misleading in daily clinical practice. Thus, re-evaluation of this potentially obsolete term is warranted.

## Supplementary Information

**Additional file 1.** SUPPLEMENTARY MATERIAL for “Prognostic implications of valvular heart disease in patients with non-valvular atrial fibrillation” Samaras et al.

## Data Availability

The datasets generated and/or analysed during the current study are not publicly available due to data availability restriction regulations but are available from the corresponding author on reasonable request.

## Abbreviations

AF: Atrial fibrillation
ESC: European society of cardiology
VHD: Valvular heart disease
sVHD: Significant valvular heart disease
AS: Atrial stenosis
AR: Atrial regurgitation
MS: Mitral stenosis
MR: Mitral regurgitation
TR: Tricuspid regurgitation
PR: Pulmonary regurgitation
NT-proBNP: N-terminal pro B-type natriuretic peptide
hs-TnT: High-sensitivity troponin-T
LAVi: Left atrial volume indexed to body surface area
IVCD: Intraventricular conduction delay
HF: Heart failure
DM: Diabetes mellitus
HR: Hazard ratio
CI: Confidence interval
