# Supplementary material for: Prognostic implications of valvular heart disease in patients with non-valvular atrial fibrillation
Source: BMC Cardiovasc Disord. 2021 Sep 18;21:453. doi: 10.1186/s12872-021-02264-3 (PMC8449469; doi:10.1186/s12872-021-02264-3)
Supplement: Supplementary file 1 — Additional file 1. SUPPLEMENTARY MATERIAL for “Prognostic implications of valvular heart disease in patients with non-valvular atrial fibrillation” Samaras et al. [file 12872_2021_2264_MOESM1_ESM.docx]

**SUPPLEMENTARY MATERIAL for “Prognostic implications of valvular heart disease in patients with non-valvular atrial fibrillation” *Samaras et al.***

**Supplemental Table S1.** ﻿Cox proportional hazard models, including moderate/severe valve lesions, for the prediction of net clinical outcomes

| **Variables** | **All-cause death or HF-hospitalization** | | | | **CV death or Stroke/SEE or Major bleeding** | | | | **CV death or HF hospitalization** | | | |
| --- | --- | --- | --- | --- | --- | --- | --- | --- | --- | --- | --- | --- |
|  | **Univariate** | | **Multivariate** | | **Univariate** | | **Multivariate** | | **Univariate** | | **Multivariate** | |
|  | **HR (95% CI)** | **p value** | **HR (95% CI)** | **p value** | **HR (95% CI)** | **p value** | **HR (95% CI)** | **p value** | **HR (95% CI)** | **p value** | **HR (95% CI)** | **p value** |
| Age (years) | 1.04 (1.03-1.05) | **<0.001** | 1.02 (1.00-1.03) | **0.015** | 1.04 (1.03-1.05) | **<0.001** | 1.02 (1.00-1.04) | **0.012** | 1.04 (1.03-1.05) | **<0.001** | 1.02 (1.00-1.03) | **0.022** |
| Gender (male) | 0.92 (0.75-1.11) | 0.373 |  |  | 0.91 (0.72-1.13) | 0.378 |  |  | 0.80 (0.64-1.00) | 0.05 |  |  |
| BMI (kg/m^2^) | 0.98 (0.96-1.00) | **0.046** |  |  | 0.99 (0.97-1.01) | 0.268 |  |  | 1.00 (0.98-1.02) | 0.855 |  |  |
| Paroxysmal | 0.68 (0.55-0.84) | **<0.001** |  |  | 0.79 (0.62-1.00) | **0.049** |  |  | 0.67 (0.53-0.87) | **0.002** |  |  |
| Persistent or permanent | 1.59 (1.31-1.94) | **<0.001** |  |  | 1.31 (1.05-1.64) | **0.016** |  |  | 1.61 (1.28-2.02) | **<0.001** |  |  |
| Hypertension | 1.29 (1.00-1.67) | 0.054 |  |  | 1.33 (0.98-1.81) | **0.064** |  |  | 1.33 (1.02-1.86) | 0.061 |  |  |
| Diabetes mellitus | 1.62 (1.33-1.97) | **<0.001** | 1.50 (1.22-1.86) | **<0.001** | 1.47 (1.17-1.84) | **0.001** | 1.35 (1.06-1.72) | **0.015** | 1.75 (1.40-2.19) | **<0.001** | 1.53 (1.20-1.96) | **0.001** |
| Heart failure | 2.78 (2.26-3.43) | **<0.001** | 1.71 (1.34-2.18) | **<0.001** | 2.70 (2.13-3.43) | **<0.001** | 1.74 (1.32-2.29) | **<0.001** | 3.25 (2.54-4.15) | **<0.001** | 1.86 (1.39-2.47) | **<0.001** |
| Chronic obstructive pulmonary disease | 1.64 (1.28-2.11) | **<0.001** |  |  | 1.40 (1.04-1.88) | **0.025** |  |  | 1.80 (1.36-2.38) | **<0.001** |  |  |
| Cardiomyopathy | 1.24 (1.10-1.39) | **<0.001** |  |  | 1.21 (1.07-1.37) | **0.003** |  |  | 1.18 (1.03-1.35) | **0.016** |  |  |
| Coronary artery disease | 1.42 (1.16-1.73) | **<0.001** |  |  | 1.39 (1.12-1.74) | **0.003** |  |  | 1.62 (1.30-2.03) | **<0.001** |  |  |
| Prior myocardial infarction | 1.76 (1.43-2.16) | **<0.001** |  |  | 1.74 (1.38-2.21) | **<0.001** |  |  | 1.96 (1.55-2.48) | **<0.001** |  |  |
| Prior cardiac arrest | 2.18 (1.30-3.65) | **0.003** | 2.59 (1.50-4.48) | **0.001** | 1.47 (0.78-2.75) | 0.235 |  |  | 2.06 (1.13-3.77) | **0.019** | 2.38 (1.26-4.52) | **0.008** |
| Non-fatal stroke or SEE | 1.30 (1.01-1.67) | **0.048** |  |  | 1.51 (1.14-1.99) | **0.004** |  |  | 1.35 (1.01-1.80) | **0.042** |  |  |
| Non-fatal major bleeding | 1.23 (0.95-1.60) | 0.123 |  |  | 1.51 (1.13-2.01) | **0.005** | 1.41 (1.05-1.91) | **0.024** | 1.20 (0.89-1.62) | 0.244 |  |  |
| eGFR at discharge (ml/min/1.73m^2^) | 0.98 (0.98-0.99) | **<0.001** |  |  | 0.99 (0.98-0.99) | **<0.001** |  |  | 0.98 (0.98-0.99) | **<0.001** |  |  |
| LVEF (%) | 0.96 (0.96-0.97) | **<0.001** | 0.98 (0.97-0.99) | **<0.001** | 0.96 (0.95-0.97) | **<0.001** | 0.97 (0.96-0.98) | **<0.001** | 0.96 (0.95-0.97) | **<0.001** | 0.98 (0.97-0.99) | **<0.001** |
| LAVi (mL/m^2^) | 1.04 (1.04-1.05) | **<0.001** | 1.03 (1.02-1.04) | **<0.001** | 1.04 (1.03-1.05) | **<0.001** | 1.03 (1.02-1.04) | **<0.001** | 1.04(1.03-1.05) | **<0.001** | 1.03 (1.02-1.04) | **<0.001** |
| NT-proBNP (pg/ml) | 1.00 (1.00-1.00) | **<0.001** | 1.00 (1.00-1.00) | **<0.001** | 1.00 (1.00-1.00) | **<0.001** | 1.00 (1.00-1.00) | **<0.001** | 1.00 (1.00-1.00) | **<0.001** | 1.00 (1.00-1.00) | **<0.001** |
| hs-TnT (pg/ml) | 1.00 (1.00-1.00) | **<0.001** | 1.00 (1.00-1.00) | **0.001** | 1.00 (1.00-1.00) | **<0.001** | 1.00 (1.00-1.00) | **<0.001** | 1.00 (1.00-1.00) | **<0.001** | 1.00 (1.00-1.00) | **0.001** |
| Vitamin K antagonist | 1.55 (1.26-1.90) | **<0.001** |  |  | 1.60 (1.27-2.02) | **<0.001** |  |  | 1.64 (1.30-2.06) | **<0.001** |  |  |
| Non-vitamin K antagonist | 0.66 (0.54-0.80) | **<0.001** |  |  | 0.60 (0.48-0.75) | **<0.001** |  |  | 0.67 (0.53-0.84) | **<0.001** |  |  |
| Antiarrhythmic agent | 0.69 (0.53-0.90) | **0.006** |  |  | 0.67 (0.51-0.89) | **0.006** |  |  | 0.67 (0.50-0.89) | **0.006** |  |  |
| Moderate/Severe AS | 2.61 (1.97-3.46) | **<0.001** | 1.87 (1.37-2.56) | **<0.001** | 2.41 (1.74-3.32) | **<0.001** | 1.56 (1.08-2.24) | **0.017** | 2.61 (1.90-3.60) | **<0.001** | 1.80 (1.26-2.57) | **0.001** |
| Moderate/Severe AR | 1.49 (1.10-2.01) | **0.009** |  |  | 1.38 (0.97-1.96) | 0.075 |  |  | 1.51 (1.08-2.12) | **0.017** |  |  |
| Moderate/Severe MR | 2.14 (1.76-2.60) | **<0.001** |  |  | 2.01 (1.61-2.50) | **<0.001** |  |  | 2.37 (1.89-2.97) | **<0.001** |  |  |
| Moderate/Severe TR | 2.21 (1.81-2.68) | **<0.001** |  |  | 2.16 (1.73-2.69) | **<0.001** |  |  | 2.32 (1.86-2.90) | **<0.001** |  |  |

Adjusted hazard ratios as displayed only if the respective variable in independently associated with risk of outcomes. VHD, valvular heart disease; CV, cardiovascular; HF, heart failure; AF, atrial fibrillation; BMI, body mass index; eGFR, estimated glomerular filtration rate; SEE, systemic embolic event; LVEF, left ventricular ejection fraction; LAVi, indexed left atrial volume; NT- proBNP, N- terminal pro-B-type natriuretic peptide; hs-TnT, cardiac troponin T measured with high- sensitivity assay; AS, aortic stenosis; AR, aortic regurgitation; MR, mitral regurgitation; TR, tricuspic regurgitation; HR, hazard ratio; CI, confidence interval


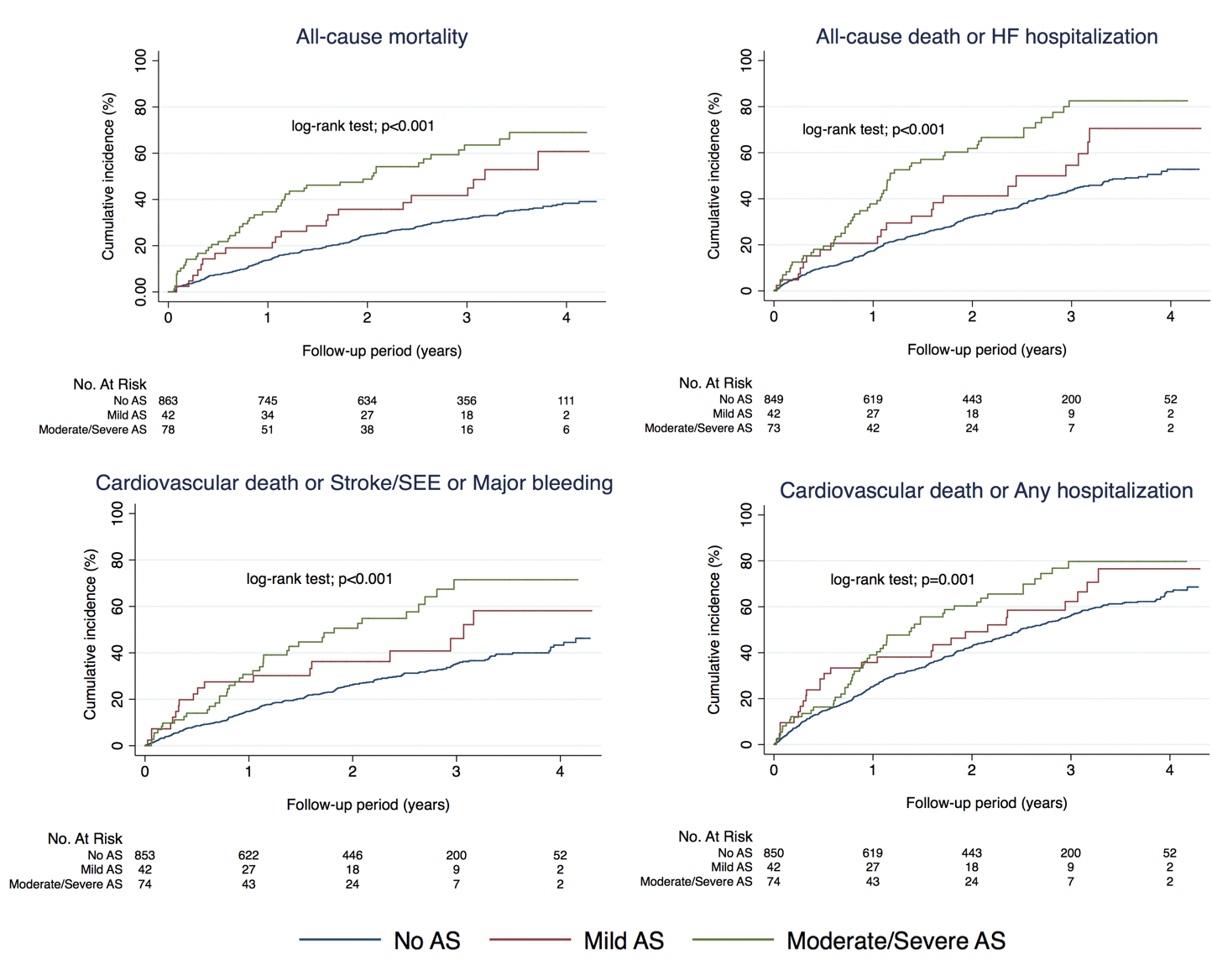


**Supplemental Figure 1**

Cumulative incidence of all-cause mortality and net clinical outcomes by presence and severity of AS. AS, aortic stenosis; HF, heart failure; SEE, systemic embolic event
